# Optoacoustic properties of Doxorubicin – A pilot study

**DOI:** 10.1371/journal.pone.0217576

**Published:** 2019-05-31

**Authors:** Melanie A. Kimm, Claudia Gross, Xose Luis Déan-Ben, Avihai Ron, Ernst J. Rummeny, Hsiao-Chun Amy Lin, Carsten Höltke, Daniel Razansky, Moritz Wildgruber

**Affiliations:** 1 Department of Diagnostic and Interventional Radiology, Technische Universität München, Munich, Germany; 2 Faculty of Medicine and Institute of Pharmacology and Toxicology, University of Zürich, Zürich, Switzerland; 3 Institute for Biomedical Engineering and Department of Information Technology and Electrical Engineering, ETH Zürich, Zürich, Switzerland; 4 Institute for Biological and Medical Imaging, Helmholtz Zentrum München and Center for Translational Cancer Research, TranslaTUM, Munich, Germany; 5 Translational Research Imaging Center, Department of Clinical Radiology, Universitätsklinikum Münster, Münster, Germany; Chinese Academy of Sciences, CHINA

## Abstract

Doxorubicin (DOX) is a widely used chemotherapeutic anticancer drug. Its intrinsic fluorescence properties enable investigation of tumor response, drug distribution and metabolism. First phantom studies *in vitro* showed optoacoustic property of DOX. We therefore aimed to further investigate the optoacoustic properties of DOX in biological tissue in order to explore its potential as theranostic agent. We analysed doxorubicin hydrochloride (Dox·HCl) and liposomal encapsulated doxorubicin hydrochloride (Dox·Lipo), two common drugs for anti-cancer treatment in clinical medicine. Optoacoustic measurements revealed a strong signal of both doxorubicin substrates at 488 nm excitation wavelength. Post mortem analysis of intra-tumoral injections of DOX revealed a detectable optoacoustic signal even at three days after the injection. We thereby demonstrate the general feasibility of doxorubicin detection in biological tissue by means of optoacoustic tomography, which could be applied for high resolution imaging at mesoscopic depths dictated by effective penetration of visible light into the biological tissues.

## Introduction

Doxorubicin hydrochloride, an anthracycline antibiotic, is one of the most commonly used anti-cancer therapeutics. The small amphiphilic and amphoteric molecule enters the cell by diffusion and subsequently exhibits cytotoxic properties. On the one hand, Dox·HCl intercalates into the DNA causing cell cycle arrest, on the other hand Dox·HCl inhibits Topoisomerase II and thereby induces cell death [1]. Additionally, the generation of reactive oxygen species leads to mitochondrial dysfunction, which, as a main side effect causes cardiotoxicity.

Fluorescence characteristics of Dox·HCl have been intensively investigated as they allow detection by various microscopic and macroscopic technologies [2]. Owing to its fluorescent features, Dox·HCl is easily detectable and also quantifiable by spectrophotometry [3]. In clinical and preclinical research, optical imaging is a widespread tool to monitor tumor behaviour during therapy [4]. So far, intravital microscopy and flow cytometry are used to follow Dox·HCl uptake within living cells [5, 6]. Similarly, *ex vivo* experiments in mice using fluorescence-based optical imaging systems have been reported [7]. Several analyses concerning fluorescence imaging and spectral shifts of incorporated Dox·HCl have been published so far [8]. Stucke-Ring et al. reported that intravenous injection of Dox·HCl led to a fast accumulation of Dox·HCl within the tumor cells with a fluorescent peak 6 hours after injection and fluorescence detection even ten days later [9]. Thus, the fluorescent properties of Doxorubicin are beneficial to investigate drug distribution and pharmacokinetics after drug application *in vivo*. However, fluorescence imaging is limited by poor tissue penetration of NIR light and decreased spatial resolution due to scattering and diffusion.

Optoacoustic imaging instead benefits from high contrast and spectral specificity of optical imaging in combination with the increased imaging depth and spatial resolution of ultrasound imaging. Optoacoustic imaging detects ultrasound waves produced by the absorption of pulsed near-infrared light (NIR) [10]. Multispectral optoacoustic tomography is able to visualize optical contrast of tissues or certain molecules without the use of exogenous contrast agents [11]. Highly perfused tumors are detectable and distinguishable from microenvironment by the different appearance of their neovasculature. In addition, differential spectral absorption characteristics can be used to detect multiple reporters simultaneously. Endogenous chromophores, which are detectable by optoacoustic imaging, include haemoglobin, melanin and lipids.

The aim of this study was to elucidate the intrinsic optoacoustic properties of DOX and evaluate optoacoustic detection of doxorubicin experimentally in tumors post mortem. We hypothesize that due to its intrinsic fluorescent properties, doxorubicin will generate a detectable optoacoustic signal which can be spatially resolved and thus reveal the biodistribution of the drug after injection into experimental tumors. This may open new opportunities to investigate drug distribution and mechanistic effects such as interaction between the drug and adjacent tumor tissue in proximity to the drug after *in vivo* application.

## Material and methods

### Animals

Procedures involving animals and their care were conducted in conformity with national and international guidelines (EU 2010/63) with approval from the local authority (Government of Upper Bavaria) and supervised by the Animal Care and Use Committee of Klinikum rechts der Isar (Munich Germany). Animals were housed in standard animal rooms (12 h light/dark cycle, 50–60% humidity, 18°C-23°C temperature, bedding material) in individually ventilated cage systems (IVC Techniplast) under specific pathogen-free conditions with free access to water and standard laboratory chow *ad libitum*. Post mortem excised tumors from two female C57Bl/6-albino mice (8–12 week-old, Charles River Laboratories, Europe) used for the pilot study were used in terms of the 3Rs guiding principles. One mouse without doxorubicin injection was used as negative control.

### In vitro experiments

Doxorubicin hydrochloride (Dox·HCl) (Teva, UK) and PEGylated, liposomal encapsulated Doxorubicin (Dox·Lipo) (Caelyx, EurimPharm, Germany) were supplied by the pharmacy of Klinikum rechts der Isar. Negative side effects of Doxorubicin hydrochloride are preventable by encapsulation of Dox·HCl in PEGylated liposomes. The altered pharmacokinetic profile of Dox·Lipo initiates a longer circulation rate and a reduced volume of distribution which makes the tumor more targetable during therapy [7, 12]. For the spectrophotometric study *in vitro* 46 μM Dox·HCl and Dox·Lipo were used. Spectrophotometric measurements of Dox·HCl was carried out at room temperature. Excitation profiles were measured in the spectral range from 400 to 800 nm. Optoacoustic profiles were performed using 1 mM Dox·HCl and Dox·Lipo in the imaging set up described in the section "Optoacoustic imaging".

### *Ex vivo* experiments

**Fig 1:**
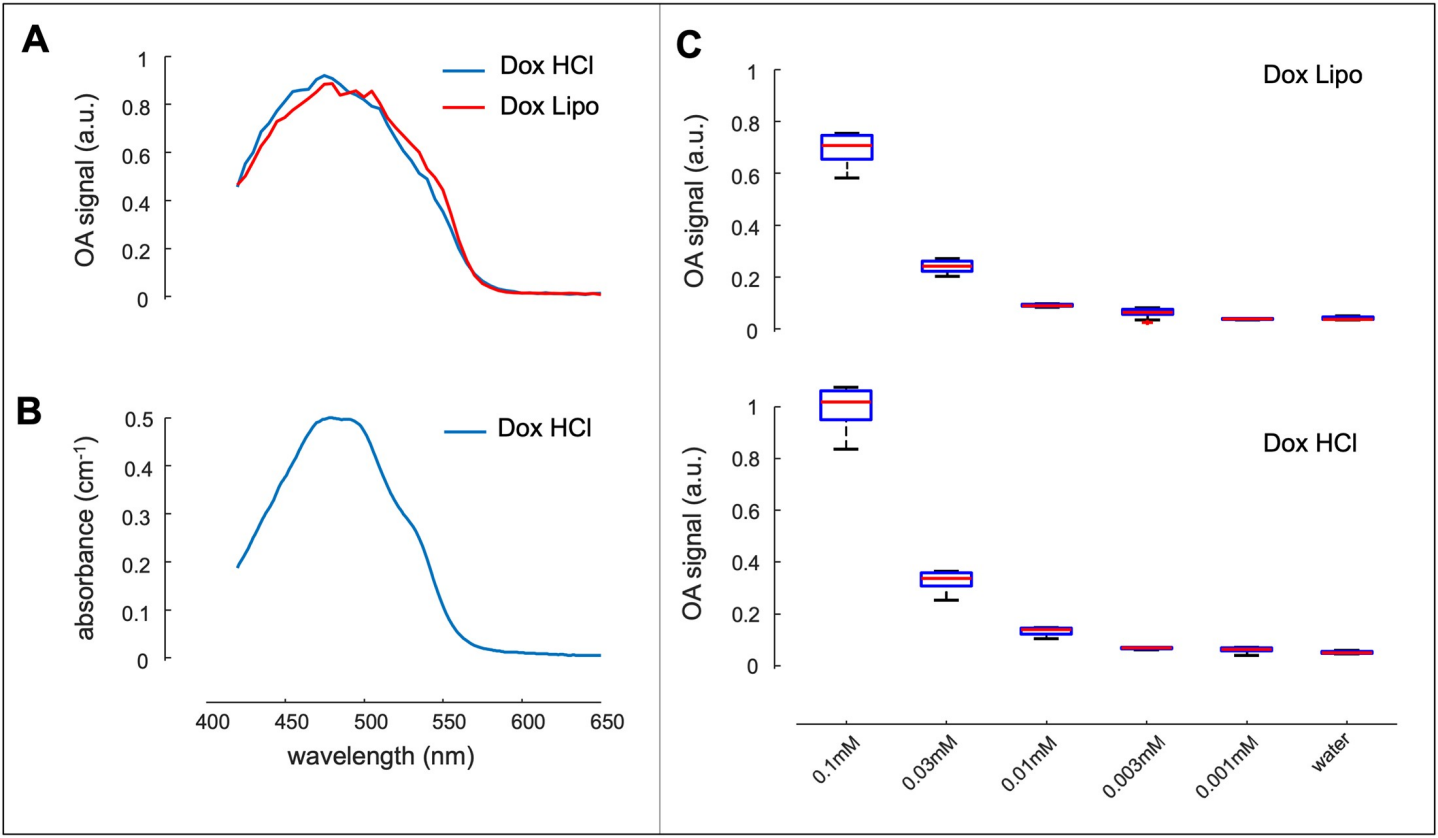
Optoacoustic properties of DOX *in vitro*. (**A**) Optoacoustic measurement of Dox·HCl and Dox·Lipo. Both formulations show a maximum peak at 480/490 nm. (**B**) Spectrophotometric assessment of Dox·HCl revealed a peak fluorescence at 480/490 nm. (**C**) Detection limit *in vitro* of Dox·HCl (lower curve) and Dox·Lipo (upper curve). For both formulations the limit was determined at 0.01 mM. Experiments were done in triplets.

We used the carcass of one CD-1 nude mouse (governmental approved vivarium of Helmholtz Zentrum Neuherberg) for post mortem s.c. injection of 1 mM, 0,1 mM and 0,01 mM Dox·HCl and Dox·Lipo in 50 μl phenol-red free matrigel (Corning). Dox·HCl and Dox·Lipo spots were injected separately s.c. in the back of the mouse (Fig 1B). Subsequently, optoacoustic imaging *ex vivo* was performed. Images of the carcass without DOX injection served as negative control.

### *In vivo* experiments

Briefly, 1x10^6 EG7 murine tumor cells (American Type Culture Collection, Manassas, USA), which are known to be Doxorubicin sensitive [13], were inoculated s.c. into the neck of two female C57Bl/6-albino mice using standard procedures and isoflurane anesthesia (1.8% Isoflurane with medical O_2_). Animals did not show any severe signs of illness following tumor formation. 5 mg Dox·Lipo per kg body weigh was injected once intratumorally. Tumor volume was measured with a caliper and tumor sizes calculated using the formula: *tumor vol [mm^3] = 0*,*5 x (length x width^2)*. The animals were euthanized at indicated time points (4 hours and 72 hours) by an injectable anesthetic overdose of ketamine-xylazine. Tumor volume was 31 mm^3 (4 hours) and 45 mm^3 (72 hours) and did not exceed the maximum tumor volume (500 mm^3). Animal protocol defined a weight loss of more than 15% compared to the weight at tumor inoculation as threshold for maximum weight loss allowed. Animals did not lose weight over time (start: 22.8 g and 24.8 g, stop: 22.7 g and 24.2 g) and no adverse events took place. Tumors were not ulcerated or blistered. No animal died due to experimental procedures or humane endpoints. Tumors were excised post mortem, washed with 1x PBS to remove excess blood and fixated in 4% neutral-buffered formalin. Until post mortem optoacoustic imaging the tumors were stored in 70% Ethanol at 4°C.

### Optoacoustic imaging

Optoacoustic imaging was performed by a custom-made spherical concave transducer array consisting of 512 ultrasound sensing elements with 5 MHz central frequency and approximately 100% bandwidth [14]. The spherical active surface of the array had a radius of 40 mm and covered an angle of 140° (solid angle 1.31π). During the measurements, the array was oriented upwards and the volume enclosed with the hemispherical cup was filled with agar to guarantee acoustic coupling and to further serve as a solid platform. The specimen lay in a supine position on top of the agar surface and optoacoustic excitation was performed with an optical parametric oscillator (OPO)-based laser guided via a fiber bundle through a central cylindrical cavity of the array. The wavelength of the laser was scanned between 440 and 640 nm (20 nm step), while the pulse repetition frequency (PRF) was set to 10 Hz. For each laser pulse, a set of 512 signals corresponding to each array element were simultaneously collected with a custom-made data acquisition system.

The measurement of DOX signal in tumor tissue *ex vivo* facilitated the quantification of optoacoustic signals generated by DOX which otherwise would be distorted by strong attenuation in the living animal and furthermore allowed a proper positioning of the tumor in the imaging system.

### Image reconstruction and spectral unmixing

Optoacoustic reconstruction of the acquired frames was performed off-line. For this purpose, the acquired raw signals were first deconvolved with the measured electrical impulse response of the detection transducer elements. The signals were then band-pass filtered with cut-off frequencies between 0.1 and 7 MHz. A three-dimensional model-based reconstruction algorithm was employed to render three dimensional images of the optical absorption distribution [15]. Specifically, a three-dimensional volume of 12×12×12 mm^3^ containing 120×120×120 voxels was reconstructed for each time instant.

The images reconstructed at multiple wavelengths were further processed to spectrally unmix the distribution of DOX within the tissue. For this, a standard linear model was used assuming that the spectral profile at any pixel is a linear combination of the optical absorbing spectra of the substances present in tissue [16].

## Results

In clinical oncology doxorubicin (DOX) is used as the pure compound doxorubicin hydrochloride (Dox·HCl) or encapsulated in liposomes (Dox·Lipo). We investigated the optoacoustic properties of both formulations first *in vitro*. 1 mM of Dox·HCl and 1 mM of Dox·Lipo were measured in an optoacoustic set up. Both formulations presented a peak at 480/490 nm (Fig 1A) which was in accordance with the literature [17].

**Fig 2:**
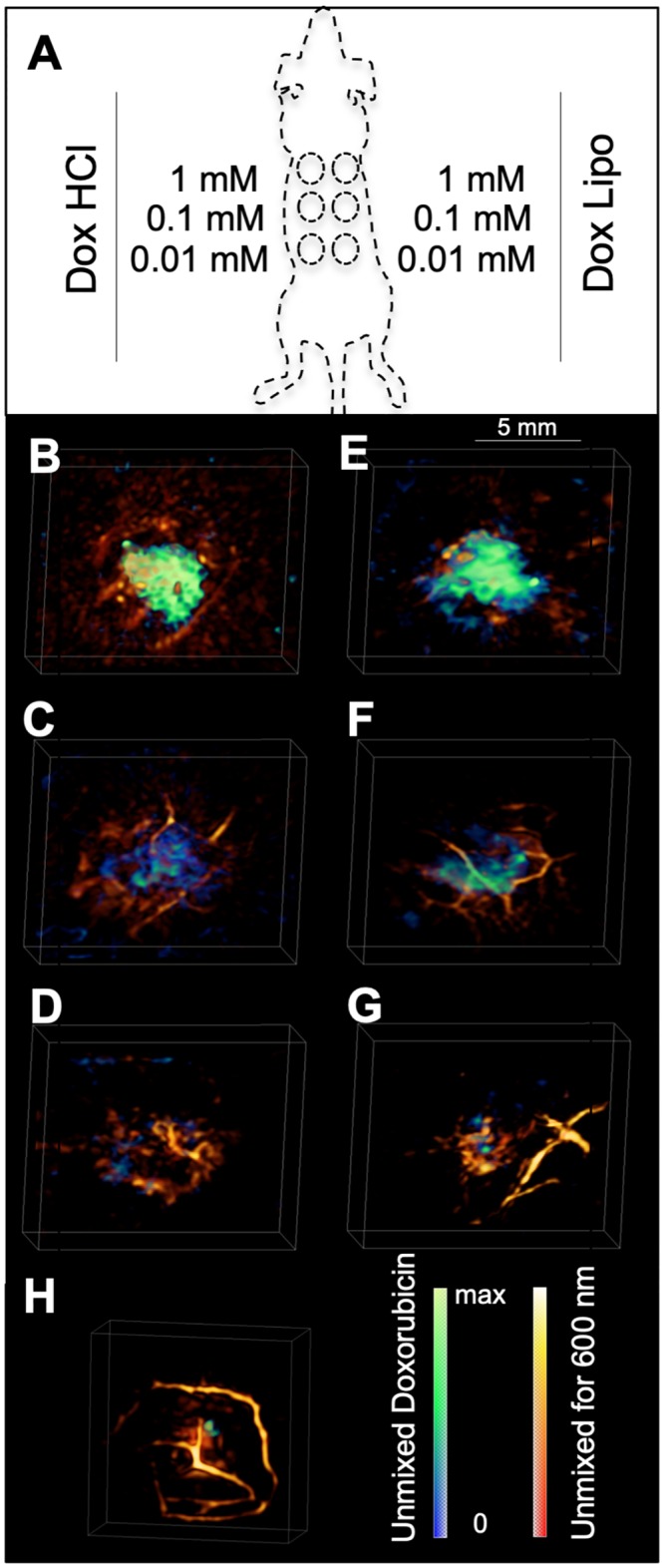
Optoacoustic properties of DOX *ex vivo*. **(A)** Schematic overview of the *ex vivo* optoacoustic imaging analysis of 1 mM (top), 0,1 mM (center) and 0,01 mM (bottom) of Dox·Lipo (right panel) and Dox·HCl (left panel). (**B-G**) Optoacoustic images of Dox·HCl (**B-D**) and Dox·Lipo (**E-G**) in 50 μl matrigel. (**B**) 1 mM Dox·HCl, (**C**) 0,1 mM Dox·HCl, (**D**) 0,01 mM Dox·HCl, (**E**) 1 mM Dox·Lipo, (**F**) 0,1 mM Dox·Lipo, (**G**) 0,01 mM Dox·Lipo, (**H**) no doxorubicin (control). Signal intensity of the unmixed DOX signal is pseudo-colored in blue/green, signal intensity of the optoacoustic signal at 600 nm is pseudo-colored in red/yellow. Scale bar = 5 mm.

We also verified the excitation profile of Dox·HCl (Fig 1B) which was in agreement with the literature. Next, we investigated the detection limit of the optoacoustic system for Dox·HCl and Dox·Lipo (Fig 1C). Both formulations decreased from 0.1 mM to 0.001 mM with a detection limit at 0.01 mM for Dox·HCl and Dox·Lipo (Fig 1C). To detect the sensitivity limit of optoacoustic imaging in a biological set up *ex vivo*, we injected 1 mM, 0,1 mM and 0,01 mM Dox·HCl and Dox·Lipo *s*.*c*. on the back of a mouse carcass (Fig 2A) and the optoacoustic signal was recorded at multiple wavelengths (440 nm to 640 nm).

Images obtained before DOX injection served as negative control (Fig 2H). Small green areas in the unmixed image associates to cross talk errors in the unmixing procedure and were considered as background signal. Images were spectrally unmixed and signal intensity pseudo-colored for better representation. The optoacoustic image at 600 nm (for anatomical features) is shown along with the unmixed signal of DOX in green to blue color bar (Fig 2B–2G). Dox·HCl exhibited good optoacoustic signal at the highest concentration used (1 mM) (Fig 2B). At a concentration of 0,1 mM DOX could still be detected (Fig 2C), with no signal detectable at 0,01 mM (Fig 2D). Similar results were found for Dox·Lipo, which rendered the strongest signal at 1 mM solution (Fig 2E) compared to 0,1 mM (Fig 2F) and 0,01 mM (Fig 2G). Tissue without any Doxorubicin content (negative control, Fig 2H) revealed minor residual contrast in the doxorubicin wavelength range, which is attributed to cross-talk of the unmixing algorithm. The concentration of 0.1 mM represents the detection limit of the *ex vivo* experiment.

**Fig 3:**
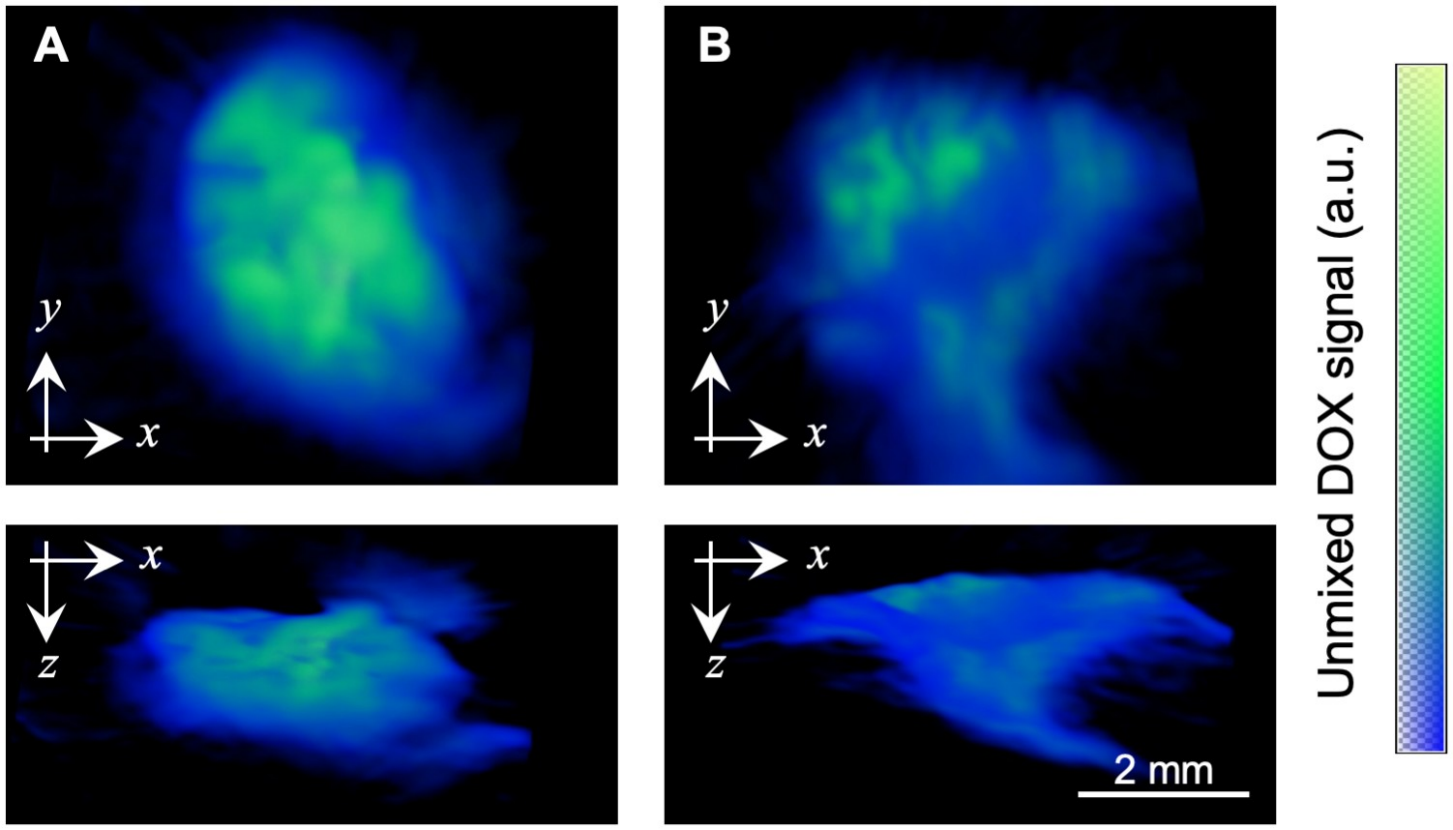
Optoacoustic detection of DOX in tumor tissue. Detection of 3.45 mM Dox·Lipo 4 hours (**A**) and 72 hours (**B**) after i.t. application. For both tumors the top and the lateral maximum intensity projections are shown. Unmixed optoacoustic signal of DOX is pseudo-coloured in blue/green. Scale bar = 2 mm.

For the optoacoustic detection of DOX in biological tissue we analyzed post mortem two tumors which were injected i.t. with 3.45 mM Dox·Lipo. Tumors were excised 4 hours (Fig 3A) and 72 hours (Fig 3B) after DOX application and formalin-fixed prior to imaging.

Since hemoglobin and deoxygenated hemoglobin are major absorbers in biological tissues with peak absorption close to that of DOX, tumors were washed to remove excess blood. After 4 hours Dox·Lipo was detected at and circular around the injection site (Fig 3A). Even 72 hours after Dox·Lipo application, DOX was still detectable (Fig 3B). Difference in signal intensity of the optoacoustic doxorubicin signal between 4h and 72h post injection was 42.5%.

## Discussion

Herein we report for the first time the investigation of the optoacoustic properties of doxorubicin in biological environment and thereby the applicability of using doxorubicin as a theranostic anti-cancer agent in combination with molecular optoacoustic imaging.

Due to its intrinsic fluorescent properties, favourable biodistribution, metabolism and drug action, doxorubicin has been examined *in vivo* and *ex vivo* by various optical techniques including multiphoton imaging [18, 19]. Similarly, fluorescence emitted by doxorubicin can be quantified *ex vivo* in fluorescence microscopy or spectrofluorometric analysis [9]. First optoacoustic investigations were reported in gelatine phantoms and in aqueous solution, showing the basic feasibility to detect doxorubicin with this technique [20]. These theranostic properties can provide novel insights into the interaction between tumor cells and doxorubicin, one of the most frequently used anti-cancer agents.

Fluorescence imaging in general is limited to investigation of superficial tissue due to diffuse attenuation and scattering of light [21]. Optoacoustic imaging combines imaging of optical contrast with ultrasound resolution. Besides macroscopic imaging of cancer biology *in vivo* in preclinical models of disease, novel optoacoustic systems are operating at the mesoscopic or microscopic scale [22, 23], which permit studying direct interactions between tumor cells, the tumor microenvironment and tumor vasculature. Label-free detection using intrinsic signalling properties is favourable over cumbersome additional labelling or additional injection of a specific imaging probe. A first label-free imaging study with breast cancer patients demonstrated the general applicability of optoacoustic imaging in the clinics. The high spatial resolution and the differentiation of oxygenated and deoxygenated haemoglobin can visualize dedicated tumor characteristics and discriminate tumorous from non-tumorous tissue [24, 25].

Combining the potential of optoacoustic imaging with the ability to visualize Doxorubicin by optoacoustic imaging can potentially allow to monitor drug distribution and metabolism *in vivo* following therapy in the preclinical setting.

Also, chemical modification of chemotherapeutics by adding signalling moieties, such as quantum dots or nanoparticles, potentially modifies biodistribution, targeting behaviour and anti-tumor efficacy. We therefore investigated the intrinsic optoacoustic properties of doxorubicin both in a phantom setup as well as in a murine tumor model to elucidate the possibility of preclinical optoacoustic imaging of therapeutic doses of doxorubicin. A first study doxorubicin containing gel phantoms revealed that concentrations down to 0.5 mg/ml can generate an optoacoustic signal, which however was not further evaluated in biological tissue [26].

Using optoacoustic imaging, we are able to show that DOX can be detected for up to 72 hours post injection, Increased permeability of the tumor vasculature allows liposomes to pass through the leaky vessels and to accumulate within the tumor tissue. In addition, longer plasma concentration and a lower clearance rate in combination with a longer half-life [27] characterizes liposomal encapsulated Dox·HCl compared to free Dox·HCl. We attribute the prolonged detection of Dox·Lipo even 72 hours after injection to the altered pharmacokinetic profile. Previous *in vitro* experiments described the time dependence of DOX accumulation in melanoma cells [28]. Cells were incubated in 5μM DOX and 10 and 30 minutes later the fluorescence intensities of 0.06 μM and 0.2μM DOX out of 10^6^ cells were detected. Those experiments reveal the potential of cellular DOX uptake shortly after drug administration. Elevated drug concentration and increased time span suggest higher intracellular uptake and accumulation of Dox·Lipo due to its altered pharmacokinetic properties. Of note, DOX uptake differs considerably between different cancer cell lines and cancer types [29].

Since DOX has absorption spectrum characteristics close to those of haemoglobin and deoxyhaemoglobin, accurate unmixing algorithms have to be applied for the analysis of *in vivo* data. Of note, the range of wavelengths where doxorubicin is able to absorb energy for acoustic conversion is far below the near infrared range (< 550 nm) and outside the biological window, restricting *in vivo* detection in deeper tissue. Micro- and meso-scopic techniques operating at superficial depths, such as raster scanning optoacoustic mesoscopy (RSOM), however could be applied to study the biodistribution of DOX *in vivo* and *ex vivo* with improved spatial resolution compared to conventional fluorescence imaging [30]. Also, dedicated methods like intravascular optoacoustic imaging or optoacoustic endoscopy may allow visualization of perivascular doxorubicin distribution in affected cancerous tissue [31, 32]. A proper calibration of the detected optoacoustic signal as a function of the concentration of DOX could serve to estimate the concentration of DOX in blood or in tissues.

Besides technological challenges it remains to be studied how the optoacoustic signal of the doxorubicin distribution in tumor tissue as detected by optoacoustic imaging correlates with the acute cytotoxic drug effects. As the kinetics of doxorubicin accumulation in tumors after intravenous [33] and intratumoural injection [34] have been investigated in detail, a follow-up study of the mechanistic drug effects in correlation to to optoacoustic signal has to prove if optoacoustic is indeed capable of monitoring anti-cancer drug effects.

For the presented experiments doxorubicin was applied according to concentrations described in the literature. Self-quenching of Dox may be an issue and has to be considered for signal quantification. Additional experiments with intravenous doxorubicin injection are needed to further assess detectability by means of optoacoustic imaging.

## Conclusion

In summary, we have shown the feasibility to map the presence of doxorubicin in biological environment of subcutaneous murine tumor xenografts by multispectral optoacoustic tomography. Different amounts of doxorubicin hydrochloride and liposomal encapsulated doxorubicin can be detected and quantified using a dedicated small animal optoacoustic imaging system. The low penetration depth of light in the used spectral range capable of effective excitation of doxorubicin, however, limits the applicability of this approach. New technologies like optoacoustic endoscopy can overcome the restriction of penetration depth in luminal biological tissue and promote its advancement towards clinical application—combining rich optical contrast and high ultrasound resolution to visualize and monitor doxorubicin delivery during therapy.

## Supporting information

S1 ARRIVE Checklist (PDF)

## References

[1] Cummings J, Willmott N, Marley EC, Smyth JF. Correlation between tumour drug disposition and the antitumour activity of doxorubicin-loaded microspheres: implications for the drugs’ in vivo mechanism of action. Biochem Pharmacol. 1993;45(12):2550–3. Epub 1993/06/22. . 8328991 10.1016/0006-2952(93)90237-q

[2] Blazkova I, Viet Nguyen H, Kominkova M, Konecna R, Chudobova D, Krejcova L, et al Fullerene as a transporter for doxorubicin investigated by analytical methods and in vivo imaging. Electrophoresis. 2014;35(7):1040–9. Epub 2013/11/21. 10.1002/elps.201300393 . 24254731

[3] Ibsen S, Su Y, Norton J, Zahavy E, Hayashi T, Adams S, et al Extraction protocol and mass spectrometry method for quantification of doxorubicin released locally from prodrugs in tumor tissue. J Mass Spectrom. 2013;48(7):768–73. Epub 2013/07/09. 10.1002/jms.3221 . 23832932 PMC4110111

[4] Ntziachristos V, Razansky D. Optical and opto-acoustic imaging. Recent Results Cancer Res. 2013;187:133–50. Epub 2012/11/28. 10.1007/978-3-642-10853-2_4 . 23179880

[5] Griffin JI, Wang G, Smith WJ, Vu VP, Scheinman R, Stitch D, et al Revealing Dynamics of Accumulation of Systemically Injected Liposomes in the Skin by Intravital Microscopy. ACS Nano. 2017;11(11):11584–93. Epub 2017/10/19. 10.1021/acsnano.7b06524 . 29045127 PMC5770233

[6] Santos MA, Goertz DE, Hynynen K. Focused Ultrasound Hyperthermia Mediated Drug Delivery Using Thermosensitive Liposomes and Visualized With in vivo Two-Photon Microscopy. Theranostics. 2017;7(10):2718–31. Epub 2017/08/19. 10.7150/thno.19662 . 28819458 PMC5558564

[7] Kress J, Rohrbach DJ, Carter KA, Luo D, Shao S, Lele S, et al Quantitative imaging of light-triggered doxorubicin release. Biomed Opt Express. 2015;6(9):3546–55. Epub 2015/09/30. 10.1364/BOE.6.003546 . 26417522 PMC4574678

[8] Motlagh NS, Parvin P, Ghasemi F, Atyabi F. Fluorescence properties of several chemotherapy drugs: doxorubicin, paclitaxel and bleomycin. Biomed Opt Express. 2016;7(6):2400–6. Epub 2016/07/05. 10.1364/BOE.7.002400 . 27375954 PMC4918592

[9] Stucke-Ring J, Ronnacker J, Brand C, Holtke C, Schliemann C, Kessler T, et al Combinatorial effects of doxorubicin and retargeted tissue factor by intratumoral entrapment of doxorubicin and proapoptotic increase of tumor vascular infarction. Oncotarget. 2016;7(50):82458–72. Epub 2016/10/16. 10.18632/oncotarget.12559 . 27738341 PMC5347705

[10] Ntziachristos V, Razansky D. Molecular imaging by means of multispectral optoacoustic tomography (MSOT). Chem Rev. 2010;110(5):2783–94. Epub 2010/04/15. 10.1021/cr9002566 . 20387910

[11] McNally LR, Mezera M, Morgan DE, Frederick PJ, Yang ES, Eltoum IE, et al Current and Emerging Clinical Applications of Multispectral Optoacoustic Tomography (MSOT) in Oncology. Clin Cancer Res. 2016;22(14):3432–9. Epub 2016/05/22. 10.1158/1078-0432.CCR-16-0573 . 27208064 PMC5046137

[12] Allen TM, Cullis PR. Liposomal drug delivery systems: from concept to clinical applications. Adv Drug Deliv Rev. 2013;65(1):36–48. Epub 2012/10/06. 10.1016/j.addr.2012.09.037 . 23036225

[13] Ehrke MJ, Verstovsek S, Maccubbin DL, Ujhazy P, Zaleskis G, Berleth E, et al Protective specific immunity induced by doxorubicin plus TNF-alpha combination treatment of EL4 lymphoma-bearing C57BL/6 mice. Int J Cancer. 2000;87(1):101–9. Epub 2000/06/22. . 10861459 10.1002/1097-0215(20000701)87:1<101::aid-ijc15>3.0.co;2-b

[14] Lin HA, Dean-Ben XL, Ivankovic I, Kimm MA, Kosanke K, Haas H, et al Characterization of Cardiac Dynamics in an Acute Myocardial Infarction Model by Four-Dimensional Optoacoustic and Magnetic Resonance Imaging. Theranostics. 2017;7(18):4470–9. Epub 2017/11/22. 10.7150/thno.20616 . 29158839 PMC5695143

[15] Ding L, Dean-Ben XL, Razansky D. Efficient 3-D Model-Based Reconstruction Scheme for Arbitrary Optoacoustic Acquisition Geometries. IEEE Trans Med Imaging. 2017;36(9):1858–67. Epub 2017/05/16. 10.1109/TMI.2017.2704019 . 28504935

[16] Razansky D, Distel M, Vinegoni C, Ma R, Perrimon N, Köster RW, et al Multispectral opto-acoustic tomography of deep-seated fluorescent proteins in vivo. Nature Photonics. 2009;3(7):412–7. 10.1038/nphoton.2009.98

[17] de Lange JH, Schipper NW, Schuurhuis GJ, ten Kate TK, van Heijningen TH, Pinedo HM, et al Quantification by laser scan microscopy of intracellular doxorubicin distribution. Cytometry. 1992;13(6):571–6. Epub 1992/01/01. 10.1002/cyto.990130604 . 1451589

[18] Carlson M, Watson AL, Anderson L, Largaespada DA, Provenzano PP. Multiphoton fluorescence lifetime imaging of chemotherapy distribution in solid tumors. J Biomed Opt. 2017;22(11):1–9. Epub 2017/12/01. 10.1117/1.JBO.22.11.116010 . 29188660 PMC5712660

[19] Dong X, Chen H, Qin J, Wei C, Liang J, Liu T, et al Thermosensitive porphyrin-incorporated hydrogel with four-arm PEG-PCL copolymer (II): doxorubicin loaded hydrogel as a dual fluorescent drug delivery system for simultaneous imaging tracking in vivo. Drug Deliv. 2017;24(1):641–50. Epub 2017/03/12. 10.1080/10717544.2017.1289570 . 28282993 PMC8241078

[20] Cook JR, Bouchard RR, Emelianov SY. Tissue-mimicking phantoms for photoacoustic and ultrasonic imaging. Biomed Opt Express. 2011;2(11):3193–206. Epub 2011/11/15. 10.1364/BOE.2.003193 . 22076278 PMC3207386

[21] Ntziachristos V, Ripoll J, Wang LV, Weissleder R. Looking and listening to light: the evolution of whole-body photonic imaging. Nat Biotechnol. 2005;23(3):313–20. Epub 2005/03/15. 10.1038/nbt1074 . 15765087

[22] Imai T, Muz B, Yeh CH, Yao J, Zhang R, Azab AK, et al Direct measurement of hypoxia in a xenograft multiple myeloma model by optical-resolution photoacoustic microscopy. Cancer Biol Ther. 2017;18(2):101–5. Epub 2017/01/04. 10.1080/15384047.2016.1276137 . 28045569 PMC5362988

[23] Wong TTW, Zhang R, Hai P, Zhang C, Pleitez MA, Aft RL, et al Fast label-free multilayered histology-like imaging of human breast cancer by photoacoustic microscopy. Sci Adv. 2017;3(5):e1602168 Epub 2017/06/01. 10.1126/sciadv.1602168 . 28560329 PMC5435415

[24] Becker A, Masthoff M, Claussen J, Ford SJ, Roll W, Burg M, et al Multispectral optoacoustic tomography of the human breast: characterisation of healthy tissue and malignant lesions using a hybrid ultrasound-optoacoustic approach. Eur Radiol. 2018;28(2):602–9. Epub 2017/08/09. 10.1007/s00330-017-5002-x . 28786007

[25] Diot G, Metz S, Noske A, Liapis E, Schroeder B, Ovsepian SV, et al Multispectral Optoacoustic Tomography (MSOT) of Human Breast Cancer. Clin Cancer Res. 2017;23(22):6912–22. Epub 2017/09/14. 10.1158/1078-0432.CCR-16-3200 . 28899968

[26] Han SH, Kang J, Wilson B, Song TK, Kim YI. The study of photoacoustic imaging without nanoparticles as a contrast agent for anti-body drug monitoring. Proc Spie. 2015;9419 Artn 94190b 10.1117/12.2082439

[27] Fan Y, Lin NM, Luo LH, Fang L, Huang ZY, Yu HF, et al Pharmacodynamic and pharmacokinetic study of pegylated liposomal doxorubicin combination (CCOP) chemotherapy in patients with peripheral T-cell lymphomas. Acta Pharmacol Sin. 2011;32(3):408–14. Epub 2011/03/05. 10.1038/aps.2010.217 . 21372831 PMC4002772

[28] Kauffman MK, Kauffman ME, Zhu H, Jia Z, Li YR. Fluorescence-Based Assays for Measuring Doxorubicin in Biological Systems. React Oxyg Species (Apex). 2016;2(6):432–9. Epub 2016/01/01. 10.20455/ros.2016.873 . 29707647 PMC5921830

[29] Tang ZH, Li T, Gao HW, Sun W, Chen XP, Wang YT, et al Platycodin D from Platycodonis Radix enhances the anti-proliferative effects of doxorubicin on breast cancer MCF-7 and MDA-MB-231 cells. Chin Med. 2014;9:16 Epub 2014/07/02. 10.1186/1749-8546-9-16 . 24982689 PMC4075934

[30] Omar M, Schwarz M, Soliman D, Symvoulidis P, Ntziachristos V. Pushing the optical imaging limits of cancer with multi-frequency-band raster-scan optoacoustic mesoscopy (RSOM). Neoplasia. 2015;17(2):208–14. Epub 2015/03/10. 10.1016/j.neo.2014.12.010 . 25748240 PMC4351295

[31] Jansen K, van Soest G, van der Steen AF. Intravascular photoacoustic imaging: a new tool for vulnerable plaque identification. Ultrasound Med Biol. 2014;40(6):1037–48. Epub 2014/03/19. 10.1016/j.ultrasmedbio.2014.01.008 . 24631379

[32] Yang JM, Maslov K, Yang HC, Zhou Q, Shung KK, Wang LV. Photoacoustic endoscopy. Opt Lett. 2009;34(10):1591–3. Epub 2009/05/19. . 19448831 10.1364/ol.34.001591 PMC2738934

[33] Laginha KM, Verwoert S, Charrois GJR, Allen TM. Determination of doxorubicin levels in whole tumor and tumor nuclei in murine breast cancer tumors. Clinical Cancer Research. 2005;11(19):6944–9. 10.1158/1078-0432.Ccr-05-0343 16203786

[34] Idani H, Matsuoka J, Yasuda T, Kobayashi K, Tanaka N. Intra-tumoral injection of doxorubicin (adriamycin) encapsulated in liposome inhibits tumor growth, prolongs survival time and is not associated with local or systemic side effects. International Journal of Cancer. 2000;88(4):645–51. 11058884 10.1002/1097-0215(20001115)88:4<645::aid-ijc20>3.0.co;2-4

